# The scenario of knowledge, attitude and practice of the Bangladeshi population towards thalassemia prevention: A nationwide study

**DOI:** 10.1371/journal.pgph.0001177

**Published:** 2022-10-21

**Authors:** Nur E. Alam, Md Shariful Islam, Md Imam Ul Khabir, Umme Suriea, Md Muzahidul Islam, Ramisa Binti Mohiuddin, Sumaiya Akter, Nahid Mahamud, Md Nazmul Islam Bappy, Dipankar Sardar, Shahin Mahmud, Kamal Chowdhury, A. K. M. Mohiuddin

**Affiliations:** 1 Department of Biotechnology and Genetic Engineering, Mawlana Bhashani Science and Technology University, Tangail, Bangladesh; 2 University of Wisconsin- Madison, Madison, Wisconsin, United States of America; 3 Department of Biological Science, Alabama State University, Montgomery, Alabama, United States of America; 4 Department of Pharmacy, Mawlana Bhashani Science and Technology University, Tangail, Bangladesh; 5 Department of Biotechnology and Genetic Engineering, Sylhet Agricultural University, Sylhet, Bangladesh; 6 Department of Biotechnology and Genetic Engineering, Khulna University, Khulna, Bangladesh; 7 Department of Biology, Claflin University, Orangeburg, South Carolina, United States of America; Sheffield Hallam University, UNITED KINGDOM

## Abstract

Thalassemia is one of the most common life-threatening yet preventable congenital hemoglobin disorders especially in South Asian regions like Bangladesh. It has become a rising public health concern for Bangladesh as 6–12% of the population are carriers and many of them are unaware of it. The purpose of the study is to inspect the knowledge and attitude towards thalassemia among the general people of Bangladesh. A cross-sectional survey was conducted in eight administrative regions of Bangladesh between January and October of 2020. A structured questionnaire was designed to collect information about thalassemia and socio-demographic characteristics. Multivariate logistic regression models were used to identify factors associated with knowledge of thalassemia. A *p*-value *<0*.*05* was considered significant. Of the 1,248 participants, only 47.4% had heard of thalassemia. Half of the participants who heard about the disease had no idea that thalassemia was not a transfusion transmitted disease. Only 49.8% of participant correctly identified consanguineous marriages as an important risk factor. Majority of them knew that marriage between two carriers can lead to a child with thalassemia major. About 72.5% knew that blood tests are a diagnosis method to determine thalassemia. Among the socio-demographic variables, the level of education of the respondents was identified as an independent predictor for knowledge (*p<0*.*05*) on thalassemia. For example, graduate (aOR: 24.88; 95% CI: 6.238–99.232) or post-graduate (aOR: 33.18; 95% CI: 7.864–140.001) participants were more aware of thalassemia than non-graduates. However, about 68.2% of the participants showed a positive attitude towards premarital screening of themselves or their family members and 85.3% were willing to donate blood to thalassemia patients. The study shows that there is a need to disseminate the information on thalassemia since the knowledge gap is huge among people. These findings will strengthen the implementation of thalassemia major awareness through educational programs, health counseling, premarital screening and campaigning.

## Introduction

Thalassemia is the most commonly inherited single-gene disorder globally that results from absence or decrease of globin chain production [1]. Depending on the nature of the mutation, there are two types of thalassemia: alpha- (α-) thalassemia and beta- (β-) thalassemia [2, 3]. High prevalence and the lack of cure make thalassemia as a global health concern [4]. Globally an estimated 100 million people are carriers of beta-thalassemia, of which approximately 100,000 are children [5]. Thalassemia is highly prevalent in Southeast Asia, the Indian subcontinent, Mediterranean, Middle Asia, Central Asia and West Africa [6]. As a consequence of migration towards non-endemic regions, thalassemia is spreading in Europe and North America [7].

South Asia (India, Pakistan, Bangladesh and Sri Lanka) is a region with a high prevalence of hemoglobinopathy, representing 23% (approximately 1.56 billion) of the world’s population [8]. Bangladesh is situated in the South Asian region, with a population of over 160 million people. About 10–19 million people of this country (6–12% of the population) carry a thalassemia gene [9]. According to World Health Organization (WHO) estimates, approximately 3% of the population (3.6 million) carries β-thalassemia and 4% (4.8 million) carries hemoglobin E (HbE) in Bangladesh [5, 10, 11]. It is assumed that over 7000 children are born with thalassemia each year in Bangladesh [12]. Furthermore, beta thalassemia or HbE has been found in 28% of assessed rural women in a recent study [13].

Allogeneic hematopoietic stem cell transplantation (alloHSCT) is currently the only curative therapy for thalassemia despite having limited access in the absence of suitable donors. However, most developing countries lack the necessary medical resources and skills to perform alloHSCT [14]. Regular blood transfusions and iron chain treatment with desferrioxamine is the standard management of thalassemia that begin in patients early in life and continue throughout their childhood, adolescence, and adult years [15]. In addition, managing thalassemia patients and living with this condition financially and emotionally for a long time constitutes a heavy burden for patients and their families [16]. Therefore, prevention is the best way to reduce the prevalence of this disorder. Different strategies were applied in different countries to reduce thalassemia. Increased awareness of general population, premarital screening and genetic counselling (PSGC) and prenatal diagnosis (PND), has led to almost total elimination of thalassemia in Cyprus and, to a considerable extent, in Greece, Italy and Sardinia [1, 17].

Although Bangladesh is in the world thalassemia belt, there is a lack of information about the epidemiology, clinical course, mortality, complications and treatment outcome of thalassemia. The general peoples of Bangladesh possess poor knowledge of the disease. This lack of awareness is influenced by region and population, including gender, marital status, education, employment, and socio-economic status [3]. Furthermore, there is no national health insurance system or organized national program in Bangladesh to raise awareness, conduct career screenings or manage thalassemia patients [9]. Therefore, this study aimed to assess public knowledge, perceptions and attitudes toward thalassemia and thalassemia screening practice.

## Materials and methods

### Study design and settings

A cross-sectional study was conducted from January to October 2020 among randomly selected 1,248 peoples (18–75 years old) in eight divisional regions (Dhaka, Chittagong, Barisal, Khulna, Rajshahi, Rangpur, Mymensingh and Sylhet) of Bangladesh. However, selection of study area and respondents in this study were based on convenience sampling. Participants were selected from different places, such as public institutions, houses and local markets in order to capture them from various backgrounds in the community. Only the people who consented were included in this study. Furthermore, those who were <18 years of age were excluded from this study.

### Questionnaire content

Data was collected via a structured questionnaire which was developed by this research team based on an extensive review of the literature [4, 9, 18, 19]. The study questionnaire was first developed in English and translated into Bengali after which translation accuracy was verified by an independent bilingual translator. Before conducting the actual data collection, the questionnaire was pilot tested in a community similar to the study population.

The questionnaire was comprised of 26 questions, and divided into four sections which included: i) demographics (9 items), ii) knowledge towards this disease (12 items), iii) attitude (3 items), and iv) practice of thalassemia screening (2 items). The responses to knowledge questions (from Q11 to Q20) were categorized into three groups: (i) correct, which included the right answers, (ii) incorrect and (iii) do not know responses included as the wrong answers. A total knowledge score was calculated by summing the responses for participants who reported having heard of thalassemia. The total score ranged between 0 and 10. According to our criteria, participants’ knowledge was considered good when the score was equal or more than 6.

Cronbach’s Alpha was used to assess the reliability coefficient which is a measure of the internal consistency of the questionnaire. The Cronbach’s alpha coefficient for knowledge and attitude questions was 0.603 where the value 0.6–0.7 is considered acceptable [20].

### Data analysis

Categorical variables were described using frequencies and percentages, and continuous variables were summarized using means and standard deviations. Socio-demographic characteristics (e.g. living place, gender, literacy status) had been considered as independent variables while ever heard of thalassemia or knowledge and attitude about thalassemia as outcome variables. Multivariable logistic regression models were generated to assess factors associated with knowledge of thalassemia. Adjusted odds ratios (aORs) and its 95% confidence intervals (CIs) were estimated. First, variables of interest were assessed using univariate analysis. Any factor that provided a univariate *p*-value ≤ 0.25 was entered into the multivariate analysis. The following variables were adjusted for in the models: age, gender, marital status, living place, literacy, occupation and socio-economic status. Collinearity was assessed using the variance inflation factor (VIF) to ensure a strong linear relationship among independent variables included in the model was not present. The goodness of fit of the model was checked using the Hosmer Lemeshow (H-L) test. *P*-values of *<0*.*05* were considered significant. Data were analyzed using IBM SPSS version 20 software.

### Ethical clearance

This study was conducted in accordance with the Declaration of Helsinki. The study was approved by the Dept. of Biotechnology and Genetic Engineering, Mawlana Bhashani Science and Technology University, Tangail-1902, Bangladesh ((Ref: MBSTU/BGE/ Research project(87)/2009/103(A)). Informed verbal and written consent were taken from each participant before starting the study and the data were duly preserved. Those who were not willing to participate, were not given the questionnaires. Confidentiality of the respondents was maintained.

## Results

### Participant’s characteristics

**Fig 1:**
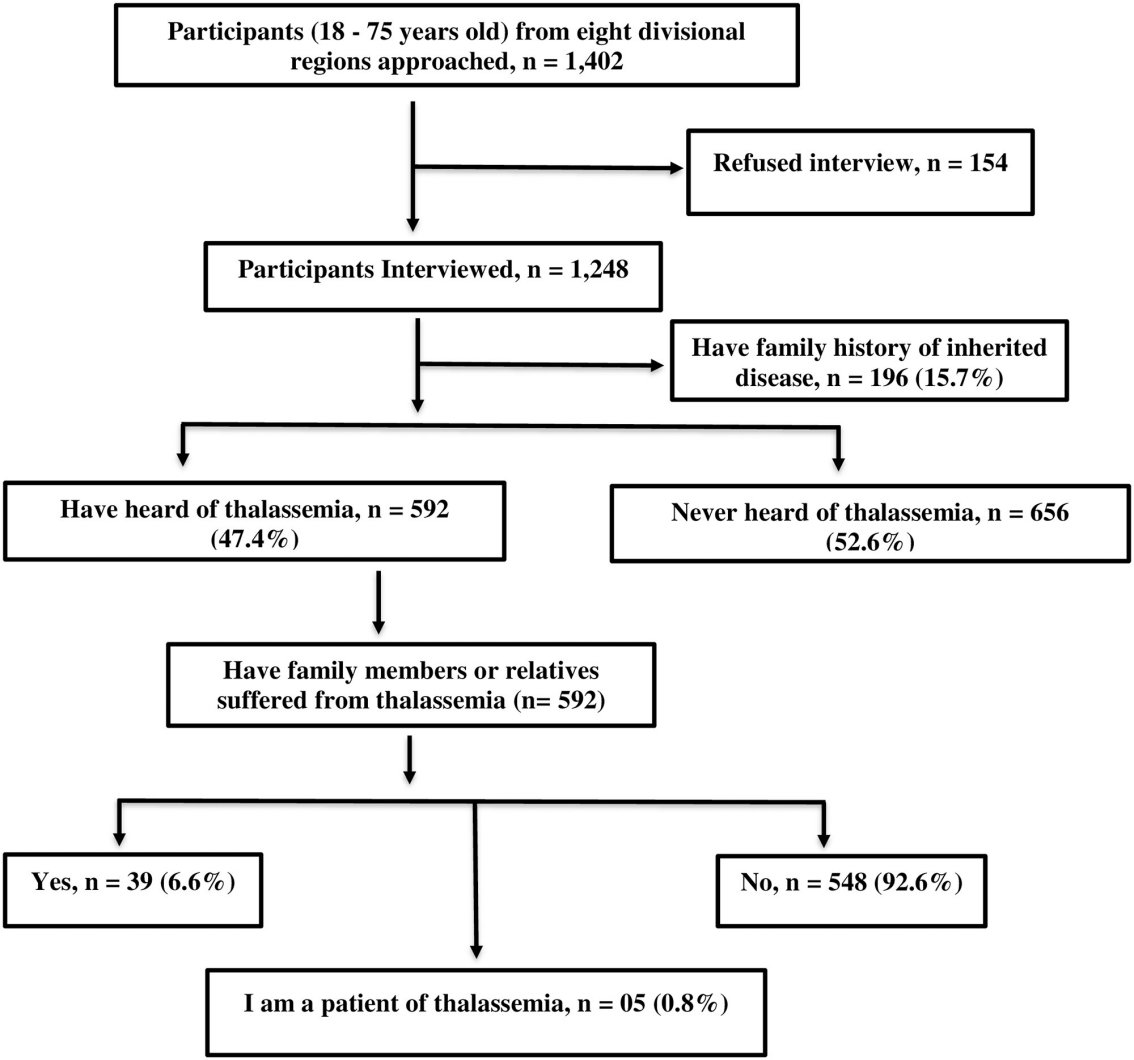
Participant’s family history of thalassemia disease.

A total of 1,248 respondents participated in the study, out of which 699 (56%) were male and remaining 549 (44%) were females. Participants’ ages ranged from 18–75 years with a mean of 37.99 ± (13.42) years. Approximately half of the participants (59.9%) were unmarried. Sixty six percent of the participants were from Semi urban/ rural community (village) while rest participating in the study were from urban areas. Of the total respondents, 33.9% were students and majority of them (82.1%) belonged to middle class families (Table 1). About 15.7% of the participants reported a family history of genetic diseases (Fig 1).

**Table 1:** Socio-demographic characteristics of respondents (n = 1,248).

Variables	n (%)
**Gender**	
Male	699 (56)
Female	549 (44)
**Marital status**	
Married	500 (40.1)
Unmarried	748 (59.9)
**Age (years)**	
18–19	39 (3.1)
20–35	615 (49.3)
36–50	382 (30.6)
51–75	212 (17)
Mean ± SD*	37.99 ± 13.42
**Living place**	
Urban	427 (34.2)
Semi-urban/rural	821 (65.8)
**Literacy status**	
Primary	380 (30.4)
Secondary	183 (14.7)
Intermediate	118 (9.5)
Undergraduate	348 (27.9)
Graduate	153 (12.3)
Post-graduate	66 (5.3)
**Occupation**	
Student	423 (33.9)
Housewife	272 (21.8)
Public sector	46 (3.7)
Private sector	139 (11.1)
Self-employed	280 (22.4)
Not employed	87 (7)
**Socio economic status**	
Low income (<15000 BDT/Month)	188 (15.1)
Middle income (15000 –<1,00,000 BDT/Month)	1024 (82.1)
High income (>1,00,000 BDT/Month)	36 (2.9)

*SD = Standard deviation

Of the total respondents, only 47.4% (592/1,248) had heard of thalassemia. These 592 participants were considered for next questions. The urban residing participants (65.1%) who had heard of thalassemia were nearly twice as high as the participants (38.2%) who lived in semi-urban or rural settings. All of the socio-demographic characteristics except gender are significant (*p*<0.001) who heard the term thalassemia in Table 2.

**Table 2:** Demographic characteristics of participants and proportion who have heard of thalassemia (n = 1,248).

Variables	Have heard of thalassemia n (%)		Chi-square (χ2)	*p* value
	No	Yes		
**Gender**			0.255	0.613
Male	363 (51.9)	336 (48.1)		
Female	293 (53.4)	256 (46.6)		
**Marital status**			437.564	**<0.001**
Unmarried	82 (16.4)	418 (83.6)		
Married	574 (76.7)	174 (23.3)		
**Living place**			81.274	**<0.001**
Urban	149 (34.9)	278 (65.1)		
Semi Town/ rural	507 (61.8)	314 (38.2)		
**Literacy status**			549.592	**<0.001**
Primary	347 (91.3)	33 (8.7)		
Secondary	143 (78.1)	40 (21.9)		
Intermediate	61 (51.7)	57 (48.3)		
Undergraduate	47 (13.5)	301 (86.5)		
Graduate	40 (26.1)	113 (73.9)		
Post-graduate	18 (27.3)	48 (72.7)		
**Employment status**			517.713	**<0.001**
Student	50 (11.8)	373 (88.2)		
Housewife	227 (83.5)	45 (16.5)		
Public sector	18 (39.1)	28 (60.9)		
Private sector	62 (44.6)	77 (55.4)		
Self-employed	234 (83.6)	46 (16.4)		
Not employed	65 (74.7)	22 (25.3)		
**Socio economic status**			21.878	**<0.001**
Lower class	128 (68.1)	60 (31.9)		
Middle class	508 (49.6)	516 (50.4)		
Upper class	24 (55.6)	16 (44.4)		

Textbooks (45.3%) was cited as the most frequently mentioned source of information about thalassemia followed by family/friends (24.8%) and internet/social media (20.6%), respectively (S1 File). Of the participants who have heard of thalassemia, 0.8% (n = 05) had thalassemia major and 6.6% (n = 39) had family members or relatives with thalassemia major (Fig 1).

### Knowledge about thalassemia

Table 3 shows the responses of participants to knowledge questions regarding thalassemia. The majority of the participants (79.1%) correctly answered that thalassemia is a hereditary disease. Only 32.8% of respondents knew that thalassemia was not a transfusion transmitted disease although half of the participants had no idea. Regarding risk factor of developing thalassemia major, 49.8% correctly identified consanguineous marriages as an important risk factor. Majority of them (69.3%) knew that marriage between two carriers can lead to a child with thalassemia major. However, 55.2% and 41.9% provide wrong answers when asked whether, the couple has a chance of having a child with thalassemia disease if one parent is a carrier and the thalassemia patients have low iron levels. Around half of the respondents (43.1%) thought that thalassemia is a completely curable disease and 72.5% knew that blood test is a diagnosis method to determine thalassemia.

**Table 3:** Knowledge about thalassemia among the participants who have heard regarding thalassemia (n = 592).

Items	Correct n (%)	Incorrect n (%)	Don’t know n (%)
Thalassemia is a hereditary disease. (Yes)	468 (79.1)	92 (15.5)	32 (5.4)
Thalassemia could be transmitted through blood transfusion from a person with thalassemia. (No)	194 (32.8)	121 (20.4)	277 (46.8)
Consanguineous marriages (marriage between close relatives) have role in the incidence of thalassemia. (Yes)	295 (49.8)	81 (13.7)	216 (36.5)
Marriage between two carriers can lead to a child with thalassemia major. (Yes)	410 (69.3)	35 (5.9)	147 (24.8)
If one parent is a carrier, the couple has a chance of having a child with thalassemia disease. (No)	99 (16.7)	327 (55.2)	166 (28)
Thalassemia is related to any of the following diseases. (Leukemia, heart problems)	192 (32.4)	104 (17.6)	296 (50.7)
Thalassemia can be identified by blood test. (Yes)	429 (72.5)	15 (2.5)	148 (25.1)
A person with thalassemia disease has low iron levels. (False)	65 (11)	248 (41.9)	279 (47.1)
Thalassemia is a curable disease. (No)	295 (49.8)	255 (43.1)	42 (7.1)
Thalassemia can be treated by (Blood Transfusion/ Iron Chelation Therapy/ Folic Acid Supplements/ Blood and Marrow Stem Cell Transplant)	392 (66.2)	-	200 (33.8)

Only 18.2% of participants have good knowledge on thalassemia. In the multivariable logistic regression model of potential predictors of knowledge on thalassemia, participants were more aware when they were in undergraduate (aOR: 23.445; 95% CI: 5.767–95.315) or they were graduate (aOR: 24.88; 95% CI: 6.238–99.232) and post-graduate (aOR: 33.18; 95% CI: 7.864–140.001). We found a significant association between overall knowledge on thalassemia and the education level of the respondents (*p*<0.001). However, 20–35 aged (189) and middle income respondents (200) were more conscious about thalassemia, but there was no statistical significance found (Table 4).

**Table 4:** Univariate and multivariate analyses of factors associated with knowledge on thalassemia (n = 1,248).

Variables	Knowledge on thalassemia		Univariate		Multivariate	
	Poor n (%)	Good n (%)	OR (95% CI)	*p* value	OR (95% CI)	*p* value
**Age**						
18–19	30 (76.9)	9 (23.1)	Ref.	**<0.001**	Ref.	0.096
20–35	426 (69.3)	189 (30.7)	1.479 (0.689–3.176)		2.031 (0.91–4.532)	
36–50	359 (94)	23 (6)	0.214 (0.091–0.503)		2.732 (0.926–8.064)	
51–75	206 (97.2)	6 (2.8)	0.097 (0.032–0.292)		1.098 (0.298–4.051)	
**Gender**						
Male	565 (80.8)	134 (19.2)	Ref.	0.311	–	**–**
Female	456 (83.1)	93 (16.9)	0.86 (0.642–1.151)		–	**–**
**Marital status**						
Unmarried	318 (63.6)	182 (36.4)	Ref.	**<0.001**	Ref.	0.111
Married	703 (94)	45 (6)	8.941 (6.287–12.715)		0.588 (0.306–1.13)	
**Living place**						
Urban	305 (71.4)	122 (28.6)	Ref.	**<0.001**	Ref.	0.183
Semi-urban/rural	716 (87.2)	105 (12.8)	0.367 (0.273–0.492)		0.788 (0.556–1.118)	
**Literacy status**						
Primary	377 (99.2)	3 (0.7)	Ref.	**<0.001**	Ref.	**<0.001**
Secondary	175 (95.6)	8 (4.4)	0.016 (0.005–0.055)		5.198 (1.33–20.269)	
Intermediate	105 (89)	13 (11)	0.091 (0.038–0.291)		10.481 (2.667–41.179)	
Undergraduate	213 (61.2)	135 (38.8)	0.248 (0.115–0.535)		23.445 (5.767–95.315)	
Graduate	107 (69.9)	46 (30.1)	1.268 (0.727–2.209)		24.88 (6.238–99.232)	
Post-graduate	44 (66.7)	22 (33.3)	0.86 (0.464–1.594)		33.18 (7.864–140.001)	
**Employment status**						
Student	258 (61)	165 (39)	Ref.	**<0.001**	Ref.	0.192
Housewife	264 (97.1)	8 (2.9)	0.47 (0.023–0.098)		0.345 (0.126–0.947)	
Public sector	36 (78.3)	10 (21.7)	0.434 (0.21–0.899)		0.696 (0.262–1.848)	
Private sector	112 (80.6)	27 (19.4)	0.377 (0.237–0.599)		0.652 (0.325–1.305)	
Self-employed	271 (96.8)	9 (3.2)	0.052 (0.026–0.104)		0.362 (0.145–0.901)	
Not employed	79 (90.8)	8 (9.2)	0.158 (0.075–0.336)		0.479 (0.193–1.189)	
**Socio economic status**						
Low income	166 (88.3)	22 (11.7)	Ref.	**0.022**	Ref.	0.09
Middle income	824 (80.5)	200 (19.5)	1.831 (1.144–2.933)		0.524 (0.285–0.963)	
High income	31 (86.1)	5 (13.9)	1.217 (0.428–3.457)		0.375 (0.114–1.237)	

### Attitude and practice towards thalassemia

In response to the questionnaire, majority of the respondents who have heard of thalassemia showed positive attitudes towards thalassemia. About 68.2% respondents would prefer premarital screening of themselves or their family members to prevent thalassemia. The majority of the participants (85.3%) had given positive response about donating blood to thalassemia patients. Likewise, 96.1% agreed to spread the information about thalassemia in their community. Respondents were asked whether they had undergone thalassemia screening process, only 7.8% reported that they had been screened for thalassemia (Table 5).

**Table 5:** Participant’s attitude and practice towards thalassemia (n = 592).

Items	Yes (n %)	No (n %)
Take any necessary steps to ensure blood testing for thalassemia before the marriage of you or your family members	404 (68.2)	188 (31.8)
Like to donate blood for thalassemia patients	505 (85.3)	87 (14.7)
Like to inform others about the potential danger of thalassemia	569 (96.1)	23 (3.9)
Ever performed a blood test for thalassemia detection	46 (7.8)	546 (92.2)

## Discussion

This study was carried out to determine the public knowledge, attitude and screening practice of thalassemia in Bangladesh. Very few studies have investigated knowledge about thalassemia in different parts of Bangladesh, and in our knowledge, this is the first study conducted among the general peoples across the country. Our study helped to address the knowledge gaps related to thalassemia.

Our study results demonstrate that only 47.4% of the respondents had heard of thalassemia. As Bangladesh lies in the thalassemia belt, the level of awareness is unexpectedly lower than the countries including Malaysia (76%), Greece (93%), Bahrain (65%) and Italy (85%) [16, 18, 21, 22]. A previous study conducted with parents of children with thalassemia in Bangladesh where 97% of respondents had never heard about the term, ‘thalassemia’ before the disease was diagnosed in their children [23]. The most worrisome finding in this study is that only 18.2% have adequate knowledge and the participants who declared to know about thalassemia, 20.4% believed that thalassemia is a transfusion transmitted disease. This result reflects a general lack of knowledge among the participants. More importantly, these participants may convey incorrect information to others who do not know about the disease.

The participants’ knowledge of thalassemia as an inherited disorder was relatively better in this study. In contrast, a study conducted in Pakistan documented that only 40% were aware of the nature of disease [24]. However, an alarming finding of our study was that half of the respondents were unaware about the role of consanguineous marriages in the incidence of thalassemia major which was incongruous with a previous study in Pakistan [19]. About 69.3% had the correct knowledge that both parents have to be carriers of beta thalassemia to have an affected child. This percentage is more than the study findings conducted in Bangladesh and Pakistan [9, 25]. Half of the respondents had misconceptions that if one parent is a carrier, a child is born with thalassemia disease. These knowledge deficits may lead to stigmatization and have profound emotional effects on thalassemia carriers.

The findings from the present study showed, respondents’ literacy status had a significant relationship with the level of knowledge on thalassemia. However, the study among participants in Bangladesh reported that thalassemia knowledge was found to be significantly related to having higher education levels. These results were consistent with the study from Kolkata and Bahrain [2, 21]. This study result showed that other socio-demographic variables like age, gender, living place were not significantly associated with knowledge of thalassemia. In contrast to our result; gender, marital status, residence and higher income were also identified as significant contributing factors of thalassemia knowledge [2].

Textbooks were selected as the most common sources of information for those who had heard about thalassemia. Similar finding was reported in a previous study in Bangladesh [9]. From this study health professionals (family doctors, obstetricians, and genetic counsellors) contributed very little to spread awareness about thalassemia. About 24.8% of respondents reported family and friends as a source of information which was similar to the result of the study performed in Italy [22]. Some studies have already revealed that physicians can play a greater role in informing the public about thalassemia. In Sardinia, 70% of the target population was informed via physicians [26].

Despite the lack of knowledge, the participants who have heard of thalassemia showed positive attitudes towards the disease. Premarital screening and genetic counseling facilities will contribute to reduce the number of babies born with thalassemia [27]. However, about 68.2% of respondents in this study agreed to do premarital screening with a blood test before marriage which is less than Oman, where 92% participants responded that they will do the test in future [28]. A very positive finding of this study was that 85.3% were willing to donate blood to transfusion-dependent thalassemia patients. This study findings raise the hopes that future awareness programs could easily increase the number of blood donors and family members with thalassemia will be able to find blood donors. Merely 7.8% of the study population who heard about the disease got themselves screened for thalassemia. This finding was similar to the Indian study where 2% of the participants performed premarital testing [24]. Ignorance, fear of being stigmatized for positive results, and endangering future prospects of getting married are considered barriers to not perform any screening test. In Bangladesh (and the South Asian region overall), marriage is synonymous with financial, emotional and physical security for many women [29].

Thalassemia is becoming a rising concern for public health in Bangladesh. Based on our study, it could be recommended that public education about thalassemia should be emphasized for successful thalassemia prevention. It has already been proven in several countries worldwide that implementation of mandatory national premarital screening programs could drastically reduce the incidence of infants born with thalassemia major [16]. However, the Ministry of Health should provide adequate training to health workers so that they can give appropriate advice in an effort to bring about behavioral change among the public to discourage consanguineous marriages. Furthermore, like the polio campaign, thalassemia prevention programs should be planned and interventions should be made all over Bangladesh to reduce the thalassemia incidence.

We recognized a few limitations in our study. Firstly, all data of this research were collected via face-to-face interviews; therefore, reporting bias due to socially desirable attitudes and behaviors might exist. Secondly, we faced some difficulties to translate the questionnaire from English to Bangla as some English words do not translate exactly into Bangla. Furthermore, a cross-sectional survey of this nature may capture only a snapshot of information about the respondents but cannot be generalized to other populations; the findings may change over time.

## Conclusion

This study has identified major areas which need to be highlighted and emphasized in rural communities and public education for thalassemia screening and awareness campaigns in Bangladesh. The Ministry of Health, Bangladesh has announced an intention to start a national screening programme for thalassemia. Our study has specifically pointed out knowledge deficits regarding the genetics and pattern of inheritance of thalassemia major. Insights of the report depict that more concise and specially designed programs for disseminating awareness regarding thalassemia should spread across the country.

## Supporting information

S1 File Major sources of information about thalassemia (n = 592). (DOCX)

S1 Questionnaire Survey questionnaire. (PDF)

S1 Data Data file. (SAV)

S2 Data Data file. (XLSX)

## References

[1] Miri-Moghaddam E, Motaharitabar E, Erfannia L, Dashipour A, Houshvar M. High School Knowledge and Attitudes towards Thalassemia in Southeastern Iran. Int J Hematol Oncol Stem Cell Res. 2014;8. 24505548 PMC3913153

[2] Basu M. A study on knowledge, attitude and practice about thalassemia among general population in outpatient department at a Tertiary Care Hospital of Kolkata. J Prev Med Holist Heal. 2015;1: 6–13.

[3] Abu-Shaheen A, Heena H, Nofal A, Abdelmoety DA, Almatary A, Alsheef M, et al. Epidemiology of thalassemia in Gulf Cooperation Council countries: A systematic review. Biomed Res Int. 2020;2020. doi: 10.1155/2020/1509501 33178817 PMC7644312

[4] Olwi DI, Merdad LA, Ramadan EK. Thalassemia: a prevalent disease yet unknown term among college students in Saudi Arabia. J Community Genet. 2018;9: 277–282. doi: 10.1007/s12687-017-0351-3 29238908 PMC6002305

[5] Chatterjee T, Chakravarty A, Chakravarty S, Chowdhury MA, Sultana R. Mutation spectrum of β-Thalassemia and other hemoglobinopathies in Chittagong, Southeast Bangladesh. Hemoglobin. 2015;39: 389–392. doi: 10.3109/03630269.2015.1078810 26402558

[6] Marengo-Rowe AJ. The Thalassemias and Related Disorders. Baylor Univ Med Cent Proc. 2007;20: 27–31. doi: 10.1080/08998280.2007.11928230 17256039 PMC1769530

[7] Peters M, Heijboer H, Smiers F, Giordano PC. Diagnosis and management of thalassaemia. BMJ. 2012;344: 1–7. doi: 10.1136/bmj.e228 22277544

[8] Black ML, Sinha S, Agarwal S, Colah R, Das R, Bellgard M, et al. A descriptive profile of β-thalassaemia mutations in India, Pakistan and Sri Lanka. J Community Genet. 2010;1: 149–157. doi: 10.1007/s12687-010-0026-9 22460247 PMC3185991

[9] Hossain MS, Hasan MM, Raheem E, Islam MS, Al Mosabbir A, Petrou M, et al. Lack of knowledge and misperceptions about thalassaemia among college students in Bangladesh: A cross-sectional baseline study. Orphanet J Rare Dis. 2020;15. doi: 10.1186/s13023-020-1323-y 32085790 PMC7035777

[10] Colah R, Gorakshakar A, Nadkarni A. Global burden, distribution and prevention of β-thalassemias and hemoglobin e disorders. Expert Rev Hematol. 2010;3: 103–117. doi: 10.1586/ehm.09.74 21082937

[11] Tahura S, Selimuzzaman M, Khan WA. Thalassaemia Prevention: Bangladesh Perspective—A Current Update. Bangladesh J Child Heal. 2017;40: 31–38. doi: 10.3329/bjch.v40i1.31553

[12] Bhuiyan R, Aklima J, Emran T, Dash R, Palit S. A study of the prevalence of thalassemia and its correlation with liver function test in different age and sex group in the Chittagong district of Bangladesh. J Basic Clin Pharm. 2012;3: 352. doi: 10.4103/0976-0105.105339 24826050 PMC3979250

[13] Merrill RD, Shamim AA, Ali H, Labrique AB, Schulze K, Christian P, et al. High prevalence of anemia with lack of iron deficiency among women in rural Bangladesh: A role for thalassemia and iron in groundwater. Asia Pac J Clin Nutr. 2012;21: 416–424. 22705433

[14] Srivastava A, Shaji R V. Cure for thalassemia major–From allogeneic hematopoietic stem cell transplantation to gene therapy. Haematologica. 2017;102: 214–223. doi: 10.3324/haematol.2015.141200 27909215 PMC5286930

[15] Ward A, Caro JJ, Green TC, Huybrechts K, Arana A, Wait S, et al. An international survey of patients with thalassemia major and their views about sustaining life-long desferrioxamine use. BMC Clin Pharmacol. 2002;2: 1–9. doi: 10.1186/1472-6904-2-3 12015817 PMC111194

[16] Wong LP, George E, Tan JAMA. Public perceptions and attitudes toward thalassaemia: Influencing factors in a multi-racial population. BMC Public Health. 2011;11: 193. doi: 10.1186/1471-2458-11-193 21447191 PMC3076274

[17] Singh L, Wade M, Agrawal M. Awareness about thalassemia and feasibility of cascade screening in families of thalassemia major patients. Int J Contemp Pediatr. 2019;6: 2526. doi: 10.18203/2349-3291.ijcp20194582

[18] Politis C, Richardson C, Yfantopoulos JG. Public knowledge of thalassemia in Greece and current concepts of the social status of the thalassemic patients. Soc Sci Med. 1991;32: 59–64. doi: 10.1016/0277-9536(91)90127-x 2008622

[19] Maheen H, Malik F, Siddique B, Qidwai A. Assessing Parental Knowledge About Thalassemia in a Thalassemia Center of Karachi, Pakistan. J Genet Couns. 2015;24: 945–951. doi: 10.1007/s10897-015-9830-z 25843562

[20] Hossain MI, Alam NE, Akter S, Suriea U, Aktar S, Shifat SK, et al. Knowledge, awareness and preventive practices of dengue outbreak in Bangladesh: A countrywide study. PLoS One. 2021;16: 1–17. doi: 10.1371/journal.pone.0252852 34111157 PMC8192001

[21] Al Hajeri A, Al Arrayed S. Public awareness of beta Thalassemia in Bahrain. Bahrain Med Bull. 2012;34.

[22] Armeli C, Robbins SJ, Eunpu D. Comparing knowledge of β-thalassemia in samples of Italians, Italian-Americans, and non-Italian-Americans. J Genet Couns. 2005;14: 365–376. doi: 10.1007/s10897-005-1123-5 16195943

[23] Hossain MS, Mahbub Hasan M, Petrou M, Telfer P, Mosabbir AA. The parental perspective of thalassaemia in Bangladesh: lack of knowledge, regret, and barriers. Orphanet J Rare Dis. 2021;16: 1–10. doi: 10.1186/s13023-021-01947-6 34271949 PMC8283743

[24] Ebrahim S, Raza AZ, Hussain M, Khan A, Kumari L, Rasheed R, et al. Knowledge and Beliefs Regarding Thalassemia in an Urban Population. Cureus. 2019;11: 1–8. doi: 10.7759/cureus.5268 31576261 PMC6764615

[25] Ishaq F, Hasnain Abid FK, Akhtar A MS. Awareness Among Parents of β-Thalassemia Major Patients, Regarding Prenatal Diagnosis and Premarital Screening. J Coll Physicians Surg Pakistan. 2012;22: 218–221. 22482376

[26] Cao A, Kan YW. The prevention of thalassemia. Cold Spring Harb Perspect Med. 2013;3: 1–15. doi: 10.1101/cshperspect.a011775 23378598 PMC3552345

[27] Cao A, Saba L, Galanello R RM. Molecular diagnosis and carrier screening for β thalassemia. Jama. 278: 1273–1277. 9333270

[28] Al Kindi R, Al Rujaibi S, Al Kendi M. Knowledge and attitude of University students towards premarital screening program. Oman Med J. 2012;27: 291–296. doi: 10.5001/omj.2012.72 23071880 PMC3464742

[29] Chattopadhyay S. ‘Rakter dosh’-corrupting blood: The challenges of preventing thalassemia in Bengal, India. Soc Sci Med. 2006;63: 2661–2673. doi: 10.1016/j.socscimed.2006.06.031 16901596

