# Supplementary material for: The scenario of knowledge, attitude and practice of the Bangladeshi population towards thalassemia prevention: A nationwide study
Source: PLOS Glob Public Health. 2022 Oct 21;2(10):e0001177. doi: 10.1371/journal.pgph.0001177 (PMC10022238; doi:10.1371/journal.pgph.0001177)
Supplement: S1 File — (DOCX) [file pgph.0001177.s001.docx]

S1 File: Major sources of information about thalassemia (n= 592).

| Sources | n | % |
| --- | --- | --- |
| Internet/ social media | 122 | 20.6 |
| Textbooks | 268 | 45.3 |
| Family/friends | 147 | 24.8 |
| Health professionals | 31 | 5.2 |
| Others | 24 | 4.1 |
