# Supplementary material for: The scenario of knowledge, attitude and practice of the Bangladeshi population towards thalassemia prevention: A nationwide study
Source: PLOS Glob Public Health. 2022 Oct 21;2(10):e0001177. doi: 10.1371/journal.pgph.0001177 (PMC10022238; doi:10.1371/journal.pgph.0001177)
Supplement: S1 Questionnaire — (PDF) [file pgph.0001177.s002.pdf]

Personal information (ব্যক্তিগত তথ্য)

1. Age (years) [বয়স (বছর)]

(i) 16 – 19 (১৬ – ১৯) (ii) 20 – 35 (২০ – ৩৫) (iii) 36 – 50 (৩৬ – ৫০) (iv) 51 – 75 (৫১ – ৭৫)

2. Region (অঞ্চল)

(i) Dhaka (ঢাকা) (ii) Barisal (বরিশাল) (iii) Chittagong (চট্টগ্রাম) (iv) Khulna (খুলনা) (v) Sylhet (সিলেট) (vi) Rajshahi (রাজশাহী) (vii) Mymensingh (ময়মনসিংহ) (viii) Rangpur (রংপুর)

3. Gender (লিঙ্গ)

(i) Male (পুরুষ) (ii) Female (মহিলা)

4. Literacy (শিক্ষাগত যোগ্যতা)

(i) Primary (প্রাথমিক) (ii) Secondary (মাধ্যমিক) (iii) Intermediate (উচ্চমাধ্যমিক) (iv) Undergraduate (স্নাতক অধ্যয়নরত) (v) Graduate (স্নাতক) (vi) Post graduate (স্নাতকোত্তর)

5. Marital status (বৈবাহিক অবস্থা)

(i) Unmarried (অবিবাহিত) (ii) Married (বিবাহিত)

6. Occupation (পেশা)

(i) Student (শিক্ষার্থী) (ii) Housewife (গৃহিণী) (iii) Public sector (সরকারি) (iv) Private sector (বেসরকারি) (v) Self-employed (স্বনির্ভর) (vi) Not employed (বেকার)

7. Living area (বাসস্থান)

(i) Urban (শহর) (ii) Semi-urban/rural (নগর/গ্রাম)

8. Socio-economic status (সামাজিক অবস্থান)

(i) Lower class (নিম্নবিত্ত) (ii) Middle class (মধ্যবিত্ত) (iii) Higher class (উচ্চবিত্ত)

9. Do you have any family history of inherited diseases? (পিতা-মাতা থেকে প্রাপ্ত কোনও রোগের পারিবারিক ইতিহাস আপনার রয়েছে কি?)

(i) Yes (হ্যাঁ) (ii) No (না)

Knowledge towards thalassemia (থ্যালাসেমিয়া সম্পর্কিত জ্ঞান)

10. Do you heard about thalassemia disease? (আপনি কি থ্যালাসেমিয়া সম্পর্কে শুনেছেন?)

(i) Yes (হ্যাঁ) (ii) No (না)

11. Is thalassemia a hereditary disease? (থ্যালাসেমিয়া কি বংশগত রোগ?)

(i) Yes (হ্যাঁ) (ii) No (না) (iii) Don't know (জানা নেই)

12. Could thalassemia be transmitted through blood transfusion from a person with thalassemia? (থ্যালাসেমিয়া আছে এমন ব্যক্তির রক্তদানের মাধ্যমে কি থ্যালাসেমিয়া ছড়াতে পারে?)

(i) Yes (হ্যাঁ) (ii) No (না) (iii) Don't know (জানা নেই)

13. Do consanguineous marriages (marriage between close relatives) have any role in the incidence of thalassemia? (নিকট আত্নীয়দের মধ্যে বিবাহ হওয়ার সাথে থ্যালাসেমিয়ার কোনো ভূমিকা আছে কি?)

(i) Yes (হ্যাঁ) (ii) No (না) (iii) Don't know (জানা নেই)

14. Marriage between two carriers can lead to a child with thalassemia major. (থ্যালাসেমিয়ার বাহকদ্বয়ের মধ্যে বিয়ে হলে তাদের সন্তানের থ্যালাসেমিয়া হতে পারে।)

(i) Yes (হ্যাঁ) (ii) No (না) (iii) Don't know (জানা নেই)

15. If one parent is a carrier, the couple has a chance of having a child with thalassemia disease. (পিতামাতার যে কোনো একজন যদি থ্যালাসেমিয়ার বাহক হয়, তবে সেই দম্পতির সন্তানের থ্যালাসেমিয়া রোগ হতে পারে।)

(i) Yes (হ্যাঁ) (ii) No (না) (iii) Don't know (জানা নেই)

16. Do you believe that thalassemia is related to any of the following diseases? (আপনার কি মনে হয় থ্যালাসেমিয়া রোগটি নিচের রোগগুলির সাথে সম্পর্কিত?)

(i) Leukemia (লিউকেমিয়া) (ii) Cancer (ক্যান্সার) (iii) Kidney failure (কিডনি বিকল) (iv) Heart problems (হৃদপিণ্ডজনিত সমস্যা) (v) All of the above (উপরের সবগুলি) (vi) Don't know (জানা নেই)

17. Thalassemia can be identified by blood test. (রক্তপরীক্ষার মাধ্যমে থ্যালাসেমিয়া চিহ্নিত করা যায়।)

(i) Yes (হ্যাঁ) (ii) No (না) (iii) Don't know (জানা নেই)

18. A person with thalassemia disease has low iron levels. (থ্যালাসেমিয়া রোগে আক্রান্ত ব্যক্তির আয়রনের মাত্রা কম থাকে।)

(i) True (সত্য) (ii) False (মিথ্যা) (iii) Don't know (জানা নেই)

19. Is thalassemia a curable disease? (থ্যালাসেমিয়া কি নিরাময় যোগ্য?)

(i) Yes (হ্যাঁ) (ii) No (না) (iii) Don't know (জানা নেই)

20. Can thalassemia be treated by (নিচের কোনটি দ্বারা থ্যালাসেমিয়ার চিকিৎসা সম্ভব)

(i) Blood Transfusion (রক্ত গ্রহণের মাধ্যমে) (ii) Iron Chelation Therapy (আয়রণ চিলেশন থেরাপি) (iii) Folic Acid Supplements (ফলিক এসিড সরবরাহ) (iv) Blood and Marrow Stem Cell Transplant (রক্ত এবং ম্যারো স্টেম সেল ট্রান্সপ্ল্যান্ট) (v) All of the above (উপরের সবগুলি) (vi) Don't know (জানা নেই)

21. Source of information (যেখান থেকে থ্যালাসেমিয়া সম্পর্কে শুনেছি)

(i) Internet/ social media (ইন্টারনেট/সামাজিক যোগাযোগ মাধ্যম) (ii) Textbooks (পাঠ্যবই) (iii) Family/friends (পরিবার/বন্ধুবান্ধব) (iv) Health professionals (স্বাস্থ্যকর্মী) (v) Other (অন্যান্য)

Attitude towards thalassemia prevention (থ্যালাসেমিয়া প্রতিরোধের প্রতি মনোভাব)

22. Do you take any necessary steps to ensure blood testing for thalassemia before the marriage of you or your family members? (আপনি বা আপনার পরিবারের কেউ কি বিবাহ বন্ধনের পূর্বে রক্ত পরীক্ষার মাধ্যমে থ্যালাসেমিয়ার যাচাই করবেন?)

(i) Yes (হ্যাঁ) (ii) No (না)

23. Do you like to donate your blood for thalassemia patients? (আপনি কি থ্যালাসেমিয়ায় আক্রান্ত রোগীকে রক্তদান করতে ইচ্ছুক?)

(i) Yes (হ্যাঁ) (ii) No (না)

24. Do you like to inform others about the potential danger of thalassemia? (থ্যালাসেমিয়ার সম্ভাব্য ঝুঁকি সম্পর্কে কি আপনি অন্যদের কে জানাবেন?)

(i) Yes (হ্যাঁ) (ii) No (না)

Practice status of respondents towards thalassemia detection (উত্তরদাতাদের থ্যালাসেমিয়া সনাক্তকরণের অনুশীলন)

25. Do you ever performed a blood test for thalassemia detection? (থ্যালাসেমিয়া সনাক্তকরণের জন্য আপনি কি কখনও রক্ত পরীক্ষা করেছিলেন?)

(i) Yes (হ্যাঁ) (ii) No (না)

26. Do you have any family member or relatives suffered from thalassemia disease? (আপনার পরিবারে বা আত্মীয়দের মধ্যে কেউ কি থ্যালাসেমিয়ায় ভুগছেন?)

(i) Yes (হ্যাঁ) (ii) No (না) (iii) I am a patient of thalassemia (আমি নিজেই থ্যালাসেমিয়ার রোগী)

Declaration of Consent:

I am willingly participating in this survey and giving consent to the utilization of this data for academic purposes only. (আমি স্বেচ্ছায় এই সমীক্ষায় অংশ নিচ্ছি এবং কেবলমাত্র একাডেমিক উদ্দেশ্যে এই তথ্য ব্যবহারে সম্মতি দিচ্ছি)
